# Protocol for high-throughput screening of SIRT7 inhibitors using fluorescent peptide technology

**DOI:** 10.1016/j.xpro.2025.103720

**Published:** 2025-03-28

**Authors:** Tian-Shu Kang, Xiaopeng Lu, Jinke Gu, Yuan Tian, Wei-Guo Zhu

**Affiliations:** 1 Shenzhen Research Institute, Shenzhen Hospital of Guangzhou University of Chinese Medicine (Futian), Shenzhen 518000, China; 2 Department of Biochemistry and Molecular Biology, International Cancer Center, Guangdong Key Laboratory of Genome Instability and Human Disease Prevention, Marshall Laboratory of Biomedical Engineering, Shenzhen University Medical School, Shenzhen 518055, China

**Keywords:** High-Throughput Screening, Protein expression and purification, Biotechnology and bioengineering

## Abstract

Fluorescent peptides combine the polypeptides with fluorescent groups organically to facilitate the measurement of enzyme activity. Here, we present a protocol to evaluate Sirtuin 7 (SIRT7) enzymatic activity by analyzing changes in luminescent signals from its substrate polypeptides. We outline the steps for SIRT7 protein purification, enzymatic reaction setup, fluorescence spectrum detection, and data analysis. Additionally, we describe two applications targeting SIRT7: high-throughput screening of SIRT7 inhibitors using microplate and the validation of potential SIRT7 inhibitors.

For complete details on the use and execution of this protocol, please refer to Kang et al.^1^

## Highlights

• Large-scale purification of His-SIRT7 recombinant proteins from *E. coli*
• Assessment of SIRT7 enzymatic activity using fluorescent peptides
• High-throughput screening for identifying potential SIRT7 inhibitors
• Determination of the IC_50_ values for tested compounds

## Abstract (editor highlights)

Publisher’s note: Undertaking any experimental protocol requires adherence to local institutional guidelines for laboratory safety and ethics.

## Graphical abstract

**Figure:**
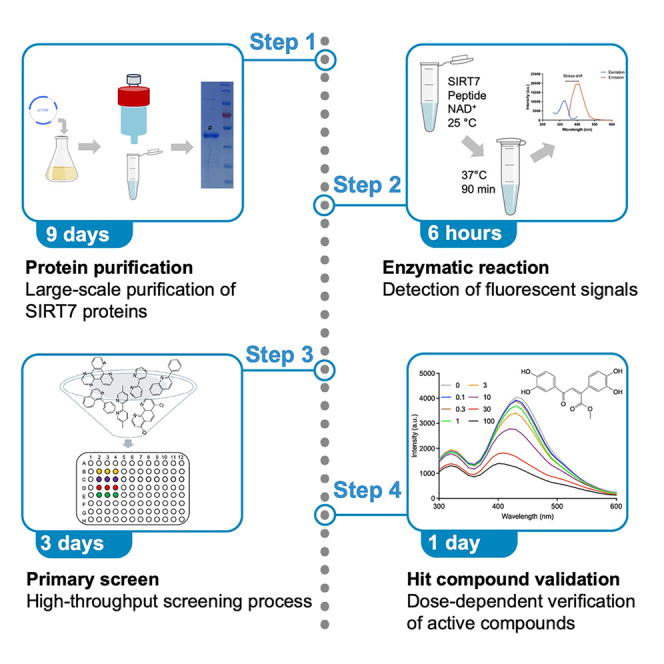

## Before you begin

SIRT7 presents great potential as a drug target for the treatment of various diseases. SIRT7 is an NAD^+^-dependent deacetylase that acts on the acetylated lysine 18 site of histone H3 (H3K18ac) that maintains the fundamental properties of cancer cell phenotypes.^2^ Additionally, coumarin and its derivatives possess excellent fluorescent properties for labeling biomacromolecules.^3^ To detect SIRT7 enzymatic activity, we modified the R group of H3_1-22_ peptides at lysine 18 with fluorophore 7-methoxycoumarin-4-acetic acid (MCA). When SIRT7, the peptides and nicotinamide adenine dinucleotide (NAD^+^) are mixed, SIRT7 protein deacylates peptides to release MCA probe that enhances fluorescent signal. Furthermore, NAD^+^ hydrolysis product nicotinamide (NAM) effectively inhibits SIRT7 enzymatic activity as the pan-sirtuins inhibitor.^4^ By using the microplate reader, we developed this luminescent system that enables rapid screening of SIRT7 inhibitors and determination of the IC_50_ values for tested compounds. Moreover, through the replacement of substrate peptides and corresponding purified enzymes, our protocol could be adapted to identify inhibitors of other epigenetic enzymes.

## Key resources table

**Table:** 

REAGENT or RESOURCE	SOURCE	IDENTIFIER
**Bacterial and virus strains**		

DH5α competent cells	TIANGEN	Cat#CB101
BL21 (DE3) competent cells	TIANGEN	Cat#CB106

**Chemicals, peptides, and recombinant proteins**		

NAD^+^	MCE	Cat#HY-B0445
NAM	Beyotime	Cat#S1761
Kanamycin	Solarbio	Cat#K8020
PageRuler prestained protein ladder	Thermo Fisher Scientific	Cat#26616
10% precast protein gel	Beyotime	Cat#P0808S
Luria-Bertani (LB) broth	Solarbio	Cat#L8291
Dimethyl sulfoxide (DMSO)	Solarbio	Cat#D8371
Dithiothreitol (DTT)	Beyotime	Cat#ST041
PMSF	MCE	Cat#HY-B0496
Protease inhibitor cocktail	MCE	Cat#HY-K0011
Sodium chloride (NaCl)	Macklin	Cat#S805281
Magnesium chloride (MgCl_2_)	Millipore	Cat#814733
Tris	Solarbio	Cat#T8060
95% ethanol	Xilong Scientific	Cat#12800101
Imidazole	Solarbio	Cat#18090
1 M Tris-HCl, pH 8.0	Solarbio	Cat#T1150
Isopropyl-β-D-thiogalactopyranoside (IPTG)	Solarbio	Cat#I8070
pET-28a	Addgene	Cat#69864-3
pET-28a-SIRT7	N/A	See Kang et al.^1^
ARTKQTARKSTGGKAPRK∗QLAGGK peptide (∗MCA)	ChinaPeptide (QYAOBIO)	N/A
SIRT7 proteins	BioVision	Cat#7876-50

**Critical commercial assays**		

Coomassie blue fast staining solution	Beyotime	Cat#P0017
Tricine-SDS-PAGE running buffer (10X)	Beyotime	Cat#P0739
TIANpure midi plasmid kit	TIANGEN	Cat#4992421

**Software and algorithms**		

GraphPad Prism software	GraphPad	https://www.graphpad.com/scientific-software/prism/
ChemDraw software	Revvity Signals	https://revvitysignals.com/products/research/chemdraw

**Other**		

HisTrap HP column	Cytiva	Cat#17524701
Superdex 75 10/300 GL	Cytiva	Cat#29148723
Unique AutoPure	Inscinstech	Cat#Unique AutoPre 25D-L1
Microplate reader	BioTek	Cat# Synergy H4 hybrid Reader
Spectrophotometer	IMPLEN	Cat#NanoPhotometer-NP80
96-well white microplate	Thermo Fisher Scientific	Cat#267350
96-well black microplate	Thermo Fisher Scientific	Cat#267342
Cell spreader	Thermo Fisher Scientific	Cat#14665231
2 L conical flask	KIMBLE	Cat#AK26505-02000
250 mL conical flask	KIMBLE	Cat#AK26505-00250
50 mL conical flask	KIMBLE	Cat#AK26505-00050
0.45 μm membrane filter	Millipore	Cat#HAWP04700
0.22 μm membrane filter	Millipore	Cat#GSWP02500
10 kDa Amicon ultra centrifugal filter	Millipore	Cat#UFC901008
Incubator shaker	Shanghai Zhichu Instrument	Cat#ZQZY-C8ES
Low-temperature centrifuge	Eppendorf	Cat#5424-R
Low-temperature centrifuge	Eppendorf	Cat#5810-R

## Materials and equipment

**Table:** Binding buffer

Reagent	Final concentration	Amount
Tris	20 mM	1.21 g
NaCl	500 mM	14.61 g
Imidazole	20 mM	0.68 g
ddH_2_O	N/A	Up to 500 mL
Total	N/A	500 mL

Filter through a 0.45 μm filter and store at 4°C for up to one month.

**CRITICAL:** Adjust to pH 8.5 at 20°C–25°C.

**Table:** Elution buffer

Reagent	Final concentration	Amount
Tris	20 mM	1.21 g
NaCl	500 mM	14.61 g
Imidazole	500 mM	17.03 g
ddH_2_O	N/A	Up to 500 mL
Total	N/A	500 mL

Filter through a 0.45 μm filter and store at 4°C for up to one month.

**CRITICAL:** Adjust to pH 8.5 at 20°C–25°C.

**Table:** Size-exclusion chromatography buffer

Reagent	Final concentration	Amount
HEPES (1 M) pH 7.5	20 mM	10 mL
NaCl	500 mM	14.61 g
ddH_2_O	N/A	Up to 500 mL
Total	N/A	500 mL

Filter through a 0.45 μm filter and store at 4°C for up to one month.

**CRITICAL:** Adjust to pH 7.5 at 20°C–25°C.

**Table:** Assay buffer for SIRT7 enzyme assay

Reagent	Final concentration	Amount
Tris-HCl (1 M), pH 8.0	10 mM	1 mL
MgCl_2_ (1 M)	4 mM	0.4 mL
Glycerol	10% (v/v)	10 mL
DTT (0.5 M)	0.2 mM	40 μL
ddH_2_O	N/A	Up to 100 mL
Total	N/A	100 mL

Filter through a 0.45 μm filter and prepare the buffer before use.

**CRITICAL:** Adjust to pH 8.0 at 20°C–25°C.

### 400 μM peptides stock solution

Dissolve 1 mg of peptides with 920 μL of ddH_2_O at 20°C–25°C, prepare 100 μL aliquots of the solution and store at −80°C for up to three months.

### 80 mM NAD^+^ solution

Dissolve 0.5 g of NAD^+^ in 10 mL of ddH_2_O, filter the solution through a 0.45 μm filter and store at −20°C for up to three months.

### 50 mg/mL kanamycin solution

Dissolve 0.5 g of kanamycin in 10 mL of dd_2_O, filter the solution through a 0.22 μm filter and store at −20°C for up to three months.

### 1 M IPTG solution

Dissolve 2.38 g of IPTG in 10 mL of ddH_2_O, filter the solution through a 0.22 μm filter and store at −20°C for up to three months.

### 3 M nicotinamide solution

Dissolve 366 mg of NAM in 1 mL of ddH_2_O and store at −20°C for up to three months.

### 10 mM tested compounds stock solutions

Dissolve tested compounds in DMSO at a concentration of 10 mM and store at −20°C for up to three months.

## Step-by-step method details

### Large-scale expression and purification of SIRT7 protein

**Timing: 9 days (3 days for step 1; 2 days for step 2; 2 days for step 3; 2 days for step 4)**

This section describes four main steps for purifying a large quantity of SIRT7 protein: (1) Amplification of SIRT7 plasmids, (2) Transformation of SIRT7 plasmids, (3) Protein expression and lysis of the cultured bacterial cells, and (4) Elution of SIRT7 protein by fast protein liquid chromatography (FPLC).

**CRITICAL:** Keep steps 1–2 operations sterile to avoid contamination, for example use gloves, 20% ethanol to disinfect surfaces, and add sterile LB medium.

1. Amplification of SIRT7 plasmids.
  a. Store a pET-28a plasmid containing the *Homo sapiens* SIRT7 gene at −20°C and thaw the plasmid on ice before use.
    ***Note:*** The human SIRT7 expression construct was kindly provided by Prof. Katrin Chua (Department of Medicine, Stanford University, Stanford, CA, USA). SIRT7 cDNA (GenBank: NM_016538.3) was cloned into pET-28a plasmid. The amino acid sequence of the his-tagged full length SIRT7 is the following:
    MGSSHHHHHHSSGLVPRGSHMASMTGGQQMGRGSEFMAAGGLSRSERKAAERVRRLREEQQRERLRQVSRILRKAAAERSAEEGRLLAESADLVTELQGRSRRREGLKRRQEEVCDDPEELRGKVRELASAVRNAKYLVVYTGAGISTAASIPDYRGPNGVWTLLQKGRSVSAADLSEAEPTLTHMSITRLHEQKLVQHVVSQNCDGLHLRSGLPRTAISELHGNMYIEVCTSCVPNREYVRVFDVTERTALHRHQTGRTCHKCGTQLRDTIVHFGERGTLGQPLNWEAATEAASRADTILCLGSSLKVLKKYPRLWCMTKPPSRRPKLYIVNLQWTPKDDWAALKLHGKCDDVMRLLMAELGLEIPAYSRWQDPIFSLATPLRAGEEGSHSRKSLCRSREEAPPGDRGAPLSSAPILGGWFGRGCTKRTKRKKVT.
  b. Store DH5α competent cells purchased from Tiangen at −80°C and keep the cells on ice before use.
  c. Add 2 μL of 300 ng/μL recombinant plasmids to 100 μL of DH5α competent cells and incubate for 30 min on ice.
  d. Place the mixture to give a heat shock in a 42°C water bath for 90 s.
  e. Transfer quickly on ice for 3 min.
  f. Add 300 μL of antibiotics-free LB medium and culture at 37°C for 1 h with shaking at 220 rpm.
  g. Spread 50 μL of the transformed cells onto a LB agar plate containing 50 μg/mL kanamycin with a cell spreader and incubate for 12–16 h at 37°C without shaking.
  h. Inoculate a single colony into 100 mL of LB liquid medium containing 50 μg/mL kanamycin and incubate for 12–16 h at 37°C with shaking at 220 rpm.
  i. Extract plasmid DNA using the TIANpure Midi Plasmid Kit.
  j. Elute plasmid DNA with 2 mL of ddH_2_O.
    ***Alternatives:*** Other plasmid backbones of the pET series could also be used, such as pET-28b and pET-28c. The instruction of TIANpure Midi plasmid kit (Tiangen, Cat#4992421) includes steps like cell lysis, plasmid binding to the column, washing, and elution. Other Midi plasmid kits (e.g., Thermo Fisher Scientific, Cat#K0481; Geneaid, Cat#PI025; or QIAGEN, Cat#12143) could also be used.
  k. Measure the plasmid concentration using a spectrophotometer.
    i. Power on the NanoPhotometer and select “Nucleic Acid” mode.
    ii. Pipette 2 μL of ddH_2_O to Nanodrop pedestal and click the “Blank“ button.
    iii. Remove the blank solution.
    iv. Pipette 2 μL of the plasmid DNA samples to Nanodrop pedestal and click the “Measure” button.
    v. Clean the Nanodrop pedestal with ddH_2_O.
      ***Note:*** Check A260/A280 ratio to assess the purity of the plasmid. A ratio of around 1.7–1.9 indicates good purity, while lower values may suggest contamination with protein or other substance.
      **Pause point:** Store the plasmids at −20°C before use.
2. Transformation of SIRT7 plasmids.
  a. Store BL21(DE3) competent cells purchased from Tiangen at −80°C and thaw the cells on ice before use.
  b. Add 2 μL of 300 ng/μL recombinant plasmids to 100 μL of BL21(DE3) competent cells and incubate for 30 min on ice.
  c. Place the mixture to give a heat shock in a 42°C water bath for 90 s.
  d. Transfer quickly on ice for 3 min.
  e. Add 300 μL of antibiotics-free LB medium and culture at 37°C for 1 h with shaking at 220 rpm.
  f. Spread 50 μL of the transformed cells onto a LB agar plate containing 50 μg/mL kanamycin with a cell spreader and incubate for 12–16 h at 37°C without shaking.

***Note:*** BL21(DE3) competent cells are mainly used for protein expression, while DH5α cells are designed for plasmid cloning and propagation.

**Figure 1:**
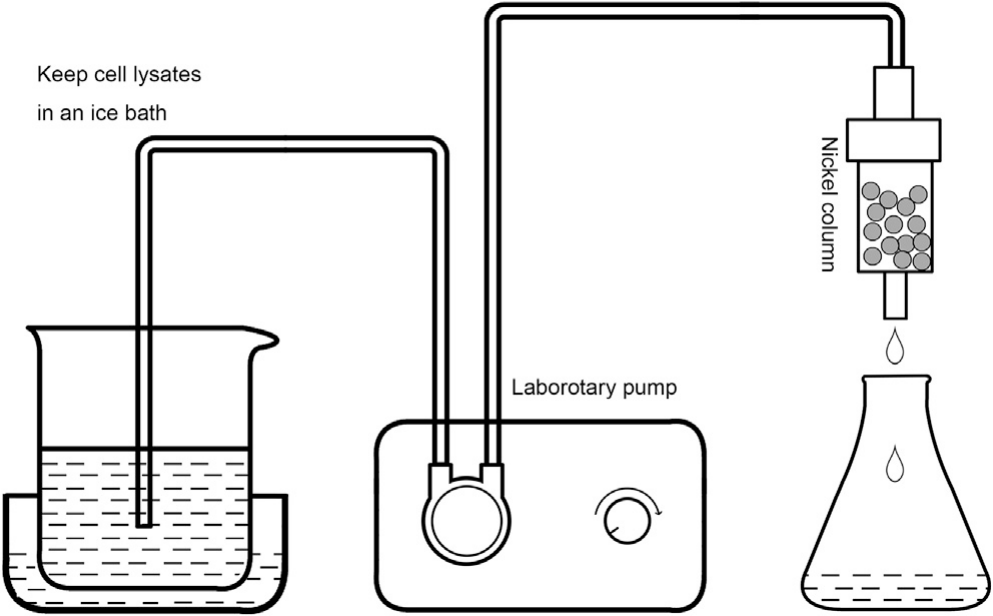
Set up the laboratory pump apparatus to combine His-SIRT7 protein into nickel column
  b. Wash HisTrap HP column with 5 column volumes of ddH_2_O.
  c. Equilibrate the column with 5 column volumes of binding buffer.
  d. Load cell lysates from step 3k onto the column and allow the waste to flow through.
  e. Attach the column to the Unique AutoPure instrument.
    ***Alternatives:*** Except Unique AutoPure instrument, using the ÄKTA pure chromatography system is also a good alternative to connect this column from the beginning of this procedure.
  f. Wash with the binding buffer until UV absorbance reaches a steady baseline.
  g. Elute with 0–100% elution buffer over 30 column volumes containing a linear gradient of imidazole.
  h. Record the UV absorbance and collect different fractions in 5 mL tubes at the UV peak (Figure 2A).
    **CRITICAL:** To prevent the binding of the host cell protein with exposed histidine to nickel column, it is crucial to add a low concentration (20 mM) of imidazole to the binding buffer.

3. Protein expression and lysis of the cultured bacterial cells.
  a. Inoculate a single transformed cell colony into 30 mL of LB medium containing 50 μg/mL kanamycin in a conical flask.
  b. Incubate the cells until OD_600_ reaches 0.6–0.8 at 37°C with shaking at 220 rpm.
    ***Note:*** When OD_600_ reaches 0.6–0.8, it indicates that the bacteria are entering the exponential growth phase.
  c. Transfer 20–30 mL of the culture to 2 L of fresh LB medium containing 50 μg/mL kanamycin.
    ***Note:*** The expansion culture ratio should ideally be between 1:50 to 1:100.
  d. Incubate the conical flask at 37°C with shaking at 220 rpm until OD_600_ reaches 0.6–0.8.
  e. Add 1 mL of IPTG stock solution to the LB medium to a final concentration of 0.5 mM.
  f. Incubate the conical flask at 18°C for 12–16 h with shaking at 220 rpm.
  g. Centrifuge the culture at 4000 x g for 10 min at 4°C and discard the supernatant.
    **Pause point:** After step 3g is finished, the harvested cell pellets can be stored at −80°C until ready for use.
  h. Resuspend the cell pellets with 150 mL of binding buffer containing 1x protease inhibitor cocktail in a beaker.
  i. Disrupt the cells using an ultra-high pressure continuous flow cell disrupter for 10 min at 100 Mpa and 4°C.
    ***Optional:*** If the suspension is very viscous, disrupt the cell suspension by sonication to break DNA, using 30 cycles of 5s ON and 5 s OFF at 60% power.
    ***Alternatives:*** Except the specified cell disrupter equipment, cell lysis can also be accomplished by other methods, for example sonication by ultrasonic homogenizer with a 6-mm microtip or enzyme digestion by lysozyme.
  j. Centrifuge the cell lysates at 20,000 x g for 30 min at 4°C and collect the supernatant containing the soluble SIRT7 protein.
  k. Filter the supernatant solution by 0.45 μm filter and collect the flowthrough liquid.
    ***Note:*** Keep the sample on ice during the process to avoid protein denaturation.
4. Elution of SIRT7 protein by fast protein liquid chromatography (FPLC).
  **CRITICAL:** Continue to keep the protein purification process in a cold environment (ice-water bath, a refrigerator, or a cold room) to avoid protein degradation.
  ***Note:*** Recommend flow rates are 5 mL/min for the 5 mL HisTrap HP column.
  a. Set up the laboratory pump apparatus (Figure 1).

**Figure 2:**
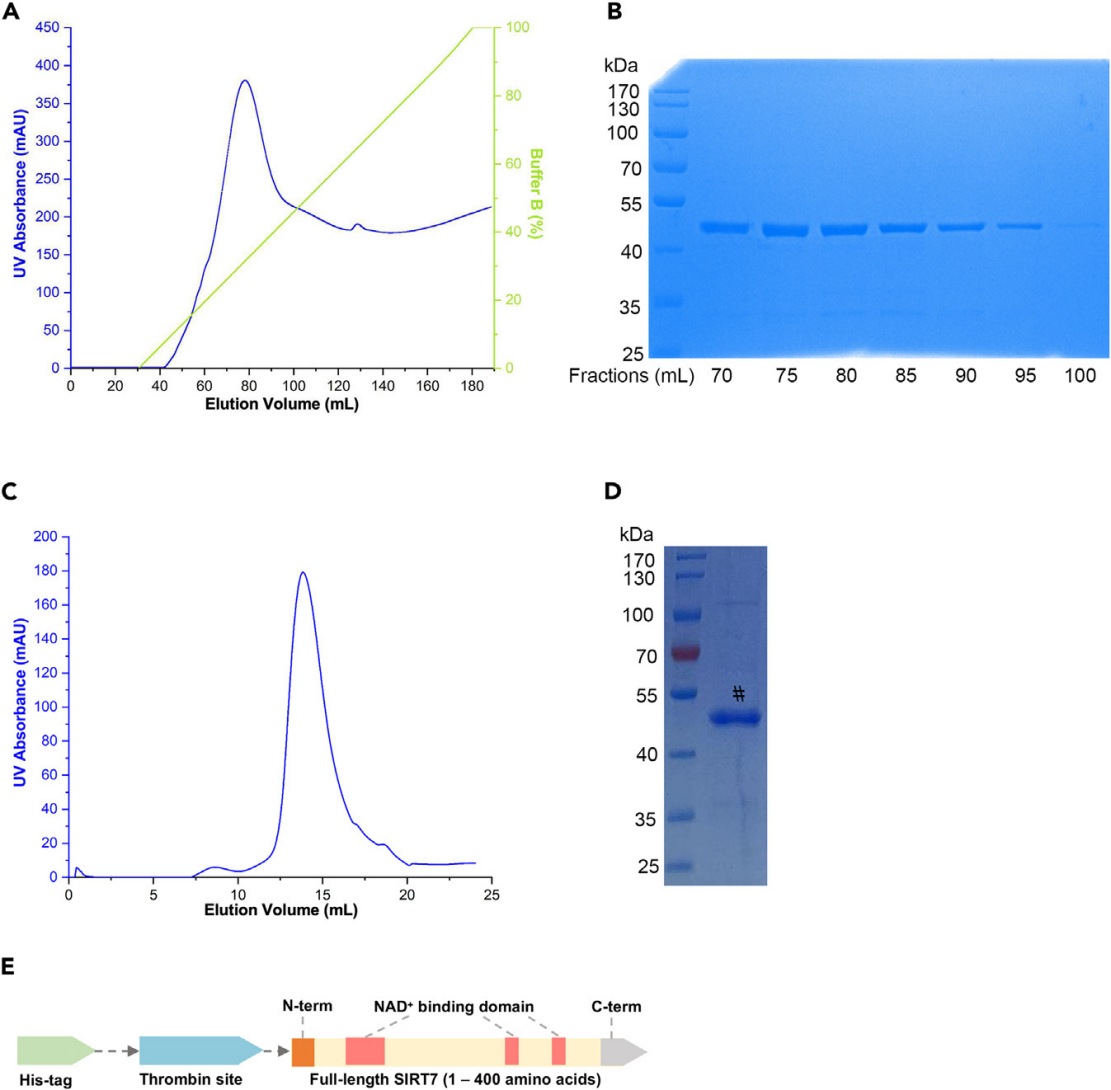
Purification of His-tagged SIRT7 protein via FPLC system (A) His-tagged SIRT7 protein is eluted using HisTrap HP column with 0–100% elution buffer. (B) 70–95 mL Fractions collected in step 4i are analyzed using SDS-PAGE and CBB staining. (C) Further purification of His-SIRT7 protein is achieved using a Superdex 75 10/300 GL size-exclusion chromatography column. (D) The purified His-SIRT7 protein in step 4p is assessed by SDS-PAGE and CBB staining. # The band of 47 kDa indicates the presence of the purified SIRT7 protein. (E) The schematic of the His-tagged recombinant full-length SIRT7 protein is shown.
  i. Perform SDS-PAGE and Coomassie brilliant blue (CBB) staining to check the protein fractions (Figure 2B).
    i. Mix 16 μL of the protein samples from different fractions with 4 μL of 5 x loading buffer.
    ii. Heat the samples at 95°C for 5 min to denature protein.
    iii. Prepare 10% precast protein gel and place the gel into the electrophoresis chamber.
      ***Note:*** The percentage of gel can be modified depending on the molecular weight of protein. In this protocol, 10% SDS-PAGE precast gel was used to detect the band of SIRT7 protein (47 kDa).
    iv. Load 4 μL of the protein ladder into the first well, followed by 10 μL of each denatured protein sample into the subsequent wells.
    v. Connect the chamber to a power supply and run the stacking gel at 90 V for 30 min and separation gel at 120 V for 90 min.
    vi. Remove the gel from the glass plates and incubate with the CBB fast staining solution for 2 h at 20°C–25°C.
    vii. Remove the gel from the staining solution and rinse fast with ddH_2_O at 50°C–60°C for 10 min.
    viii. Capture the images using a Chemiluminescent imager.
  j. Transfer His-SIRT7 eluates into Amicon ultra centrifugal filter.
  k. Centrifuge the device at 7000 x g for 30 min at 4°C until the final volume of less than 2 mL.
  l. Transfer the concentrated protein solution into the syringe.
  m. Attach the syringe and Superdex 75 10/300 GL column to the FPLC system.
  n. Equilibrate Superdex 75 10/300 GL column with 1.5 column volumes of size-exclusion chromatography buffer.
  o. Inject the syringe into the above column and perform size-exclusion chromatography to further purify SIRT7 protein (Figure 2C).
  p. Leave small fraction from step 4o and analyze the protein purity by SDS-PAGE and CBB staining (Figure 2D).
  q. Collect the peak fractions between 75-85 mL and concentrate the eluted SIRT7 protein by Amicon ultra centrifugal filter until to the concentration of 6–10 mg/mL (troubleshooting problem 1).
  r. Measure the plasmid concentration using a spectrophotometer.
    i. Power on the NanoPhotometer and select “Protein UV” mode.
    ii. Pipette 2 μL of the buffer to Nanodrop pedestal and click the “Blank” button.
    iii. Remove the buffer solution.
    iv. Pipette 2 μL of the SIRT7 protein sample to Nanodrop pedestal and click the “Measure” button.
    v. Clean the NanoDrop pedestal with the ddH_2_O.
      ***Note:*** The schematic of the His-tagged recombinant full-length SIRT7 protein is shown in Figure 2E.
      **Pause point:** Store SIRT7 protein in 50 μL of 8.5 mg/mL aliquots, snap-freeze in liquid nitrogen, and then transfer the aliquots to −80°C for storage.

### Perform SIRT7 enzyme assay using the fluorescent peptide

**Timing: 6 h (30 min for step 5; 1 h for step 6; 4.5 h for step 7)**

This section outlines the procedure for performing SIRT7 enzyme assay, which consists of three main steps: (5) stock solution preparation; (6) equipment preparation; and (7) fluorescence spectrum collection and data analysis.

5. Stock solution preparation.
  a. Dilute NAD^+^ with assay buffer to a final concentration of 5 mM.
  b. Dilute the peptides stock solution with assay buffer to a concentration of 50 μM.
    ***Note:*** The peptides were synthesized by ChinaPeptide (QYAOBIO), and characterized by high performance liquid chromatography (HPLC) analysis (Figure 3A) and mass spectrum (MS) analysis (Figure 3B). The peptide powder was dissolved in ddH_2_O to a concentration of 400 μM and stored at −80°C for up to three months.

**Figure 3:**
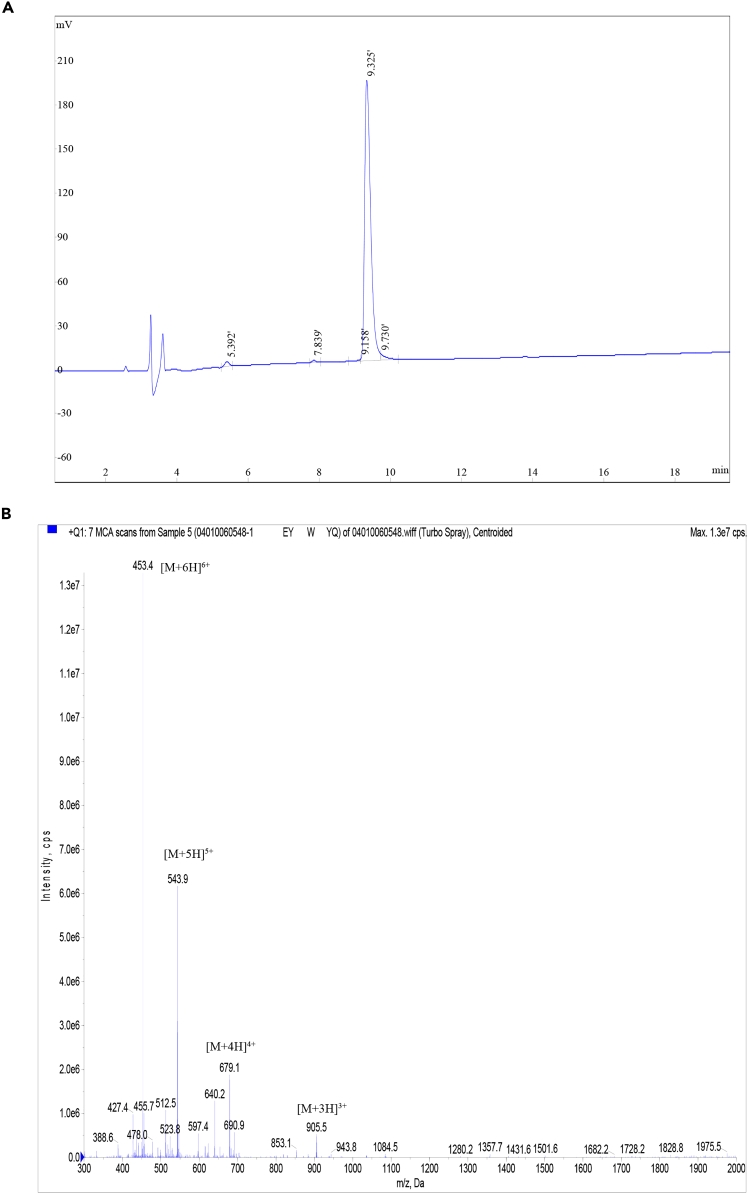
Confirmation of the synthesized fluorescent peptides (A) HPLC chromatograms of the peptides with a UV detection at wavelength of 220 nm. (B) The corresponding MS traces of the peptides.
  c. Thaw His-SIRT7 protein from step 4q on ice and dilute them with assay buffer to achieve a concentration range of 0–2.8 μg/μL.
6. Equipment preparation.
  a. Before use, turn on the microplate reader to warm up for 30 min.
  b. Connect the host computer and run the fluorescence data acquisition software.
  c. Click “New” in the task manager and select fluorescence in the detection of mode.
  d. Set the excitation wavelength to 260 nm and configure the emission spectrum detection range from 300 to 650 nm.
7. Fluorescence spectrum collection and data analysis.
  a. Add 5 μL of 5 mM NAD^+^ stock solution to the wells of the microplate.
  b. Add 5 μL of 50 μM peptide stock solution to the wells.
  c. Add 5 μL of 0–2.8 μg/μL SIRT7 protein stock solution to the wells.
  d. Add 35 μL of assay buffer to make the total volume to 50 μL (Table 1).

**Table 1:** The experimental groups were setup in SIRT7 enzyme assay

Tubes	1	2	3	4	5	6	7	8
SIRT7 (μg)	0	2	4	6	8	10	12	14
Peptides (μM)	10	10	10	10	10	10	10	10
NAD^+^(mM)	0.5	0.5	0.5	0.5	0.5	0.5	0.5	0.5
Assay buffer (μL)	35	35	35	35	35	35	35	35
Total (μL)	50	50	50	50	50	50	50	50
  e. Incubate the microplate at 37°C for 90 min in the dark to allow the reaction to proceed.
    ***Note:*** The selection of a 96-well microplate is essential for this assay, for example the white microplate (Thermo Fisher Scientific Cat#267350) or the black microplate (Thermo Fisher Scientific Cat#267342). Ensure that a new microplate is used, as fingerprints or residues can cause variations in readings.
    ***Note:*** Mix all solutions gently to avoid introducing any air bubbles.
  f. Read the plate and export the data after each reading (troubleshooting problems 2 and 3).
  g. Analyze the fluorescence intensity emission spectra (Figure 4A).
    i. Create an XY table in a spreadsheet using GraphPad Prism software.
    ii. Enter the emission wavelength range in column X and the fluorescent signals of the emission spectrum in column Y.
    iii. Click on the graph to adjust the axis format and output the results.

**Figure 4:**
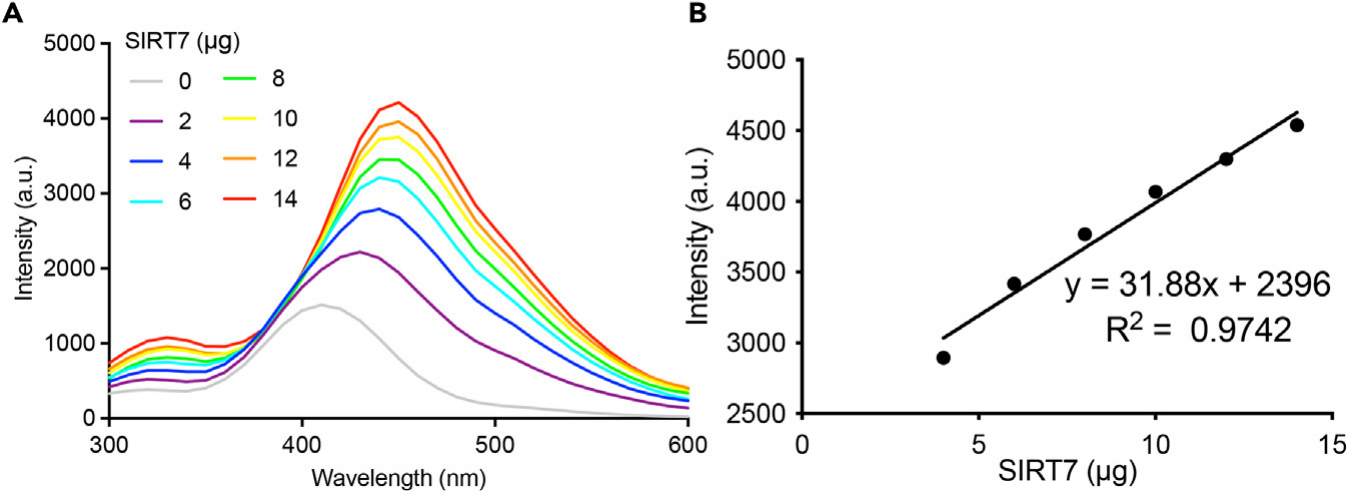
Emission spectra are measured to evaluate SIRT7 enzymatic activities (A) The fluorescence intensity emission spectra show the enzymatic activities of SIRT7 in the presence of peptide substrates and NAD+. (B) A linear relationship is observed between luminescence intensity (λ = 450 nm) and SIRT7 concentrations.
  h. Calculate the linear relationship between the luminescent intensities at 450 nm and SIRT7 concentrations (Figure 4B).
    i. Create an XY table in a Microsoft Excel spreadsheet.
    ii. Enter the emission wavelength range in column X and enter the fluorescent signals at λ = 450 nm in Column Y.
    iii. Generate a graph and draw the trendline.
    iv. Calculate the equation of the trendline and the R-squared value to assess the correlation.

### Applications of SIRT7 enzyme assay

**Timing: 4 days (3 days for step 8; 1 day for step 9)**

This section introduces two applications of SIRT7 enzyme assay.

**Figure 5:**
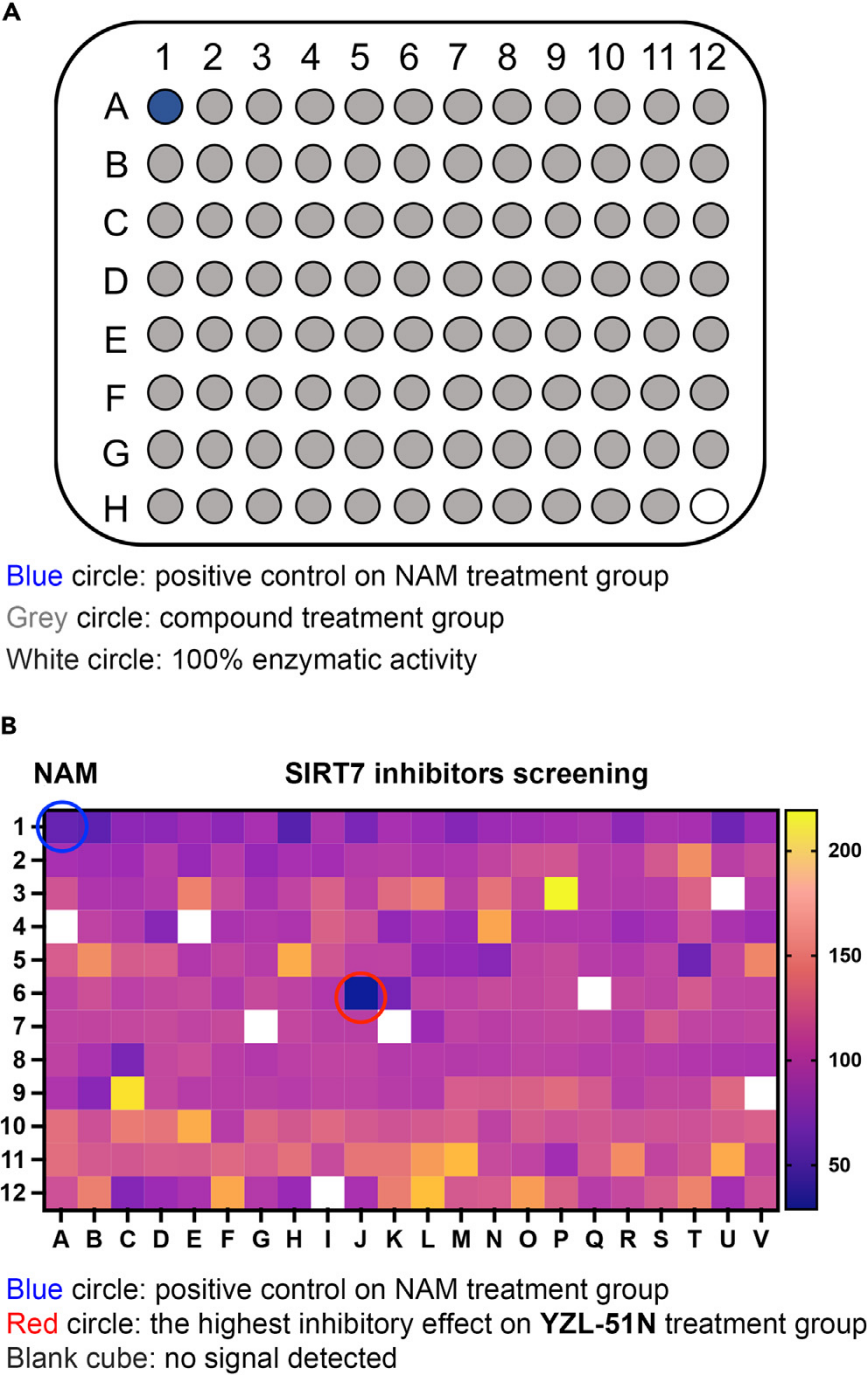
High-throughput screening for SIRT7 inhibitors (A) A 96-well microplate is designed for high-throughput screening as outlined. (B) The heatmap shows that compounds from the preserved library affect SIRT7 enzymatic activities.
  h. Incubate the microplate at 37°C for 90 min in the dark to allow the reactions to occur.
  i. Select the end-point kinetics mode in the test method.
  j. Set the excitation wavelength of 260 nm and the emission wavelength of 450 nm to capture the fluorescent signals.
  k. Calculate SIRT7 inhibition ratios for different compounds and export the heatmap (Figure 5B, troubleshooting problem 4).

***Note:*** The most potential compound **YZL-51N** (marked by the red circle) exhibits a 71.1% inhibitory effect on SIRT7 activity at a concentration of 50 μM. In contrast, NAM shows a 44.4% inhibitory effect at a centration of 3 mM^1^.

8. Application 1: High-throughput screening for SIRT7 inhibitors from the preserved compound library.
  a. Dilute NAM with assay buffer to a concentration of 30 mM.
  b. Dilute the stock solutions of tested compounds with assay buffer to a concentration of 500 μM.
  c. Add 5 μL of 5 mM NAD^+^ stock solution to the wells of the microplate.
  d. Add 5 μL of 50 μM peptide stock solution to the wells.
  e. Add 5 μL of 2.4 μg/μL SIRT7 protein stock solution to the wells.
  f. Add 5 μL of 500 μM tested compounds or 30 mM NAM to the respective wells.
  g. Add 30 μL of assay buffer to achieve a total volume of 50 μL (Figure 5A).

9. Application 2: Determination of IC_50_ of test compounds.
  a. Prepare a 1:2 dilution of compound **YZL-51N** with assay buffer starting from a concentration of 1 mM (Table 2).

**Table 2:** The **YZL-51N** treatment groups were setup in SIRT7 enzyme assay

Tubes	1	2	3	4	5	6	7	8
**YZL-51N** (μM)	0	0.1	0.3	1	3	10	30	100
SIRT7 (μg)	12	12	12	12	12	12	12	12
Peptides (μM)	10	10	10	10	10	10	10	10
NAD^+^ (mM)	0.5	0.5	0.5	0.5	0.5	0.5	0.5	0.5
Assay buffer (μL)	30	30	30	30	30	30	30	30
Total (μL)	50	50	50	50	50	50	50	50
  b. Prepare a 1:2 dilution of NAM with assay buffer starting from a concentration of 300 mM (Table 3).

**Table 3:** The NAM treatment groups were setup in SIRT7 enzyme assay

Tubes	1	2	3	4	5	6	7	8	9	10	11
NAM (mM)	0	0.001	0.003	0.01	0.03	0.1	0.3	1	3	10	30
SIRT7 (μg)	12	12	12	12	12	12	12	12	12	12	12
Peptides (μM)	10	10	10	10	10	10	10	10	10	10	10
NAD^+^ (mM)	0.5	0.5	0.5	0.5	0.5	0.5	0.5	0.5	0.5	0.5	0.5
Assay buffer (μL)	30	30	30	30	30	30	30	30	30	30	30
Total (μL)	50	50	50	50	50	50	50	50	50	50	50
  c. Add 5 μL of 5 mM NAD^+^ stock solution to the wells of the microplate.
  d. Add 5 μL of 50 μM peptide stock solution to the wells.
  e. Add 5 μL of 2.4 μg/μL SIRT7 protein stock solution to the wells.
  f. Calculate the IC_50_ of tested compounds (troubleshooting problem 5).
    i. Create an XY table in a spreadsheet using GraphPad Prism Version software.
    ii. Enter the different doses of compounds in column X and the corresponding fluorescent signals at λ = 450 nm in Column Y.
    iii. Click “Analyze” and transform the X values using Log (X).
    iv. Fit a curve using the nonlinear regression to determine the relationship between concentration and inhibition.
    v. Select the dose-response inhibition equation to model the data.
    vi. Record the IC_50_ value of **YZL-51N** (Figure 6A) and NAM (Figure 6B) according to the best-fit values in the table of results.

**Figure 6:**
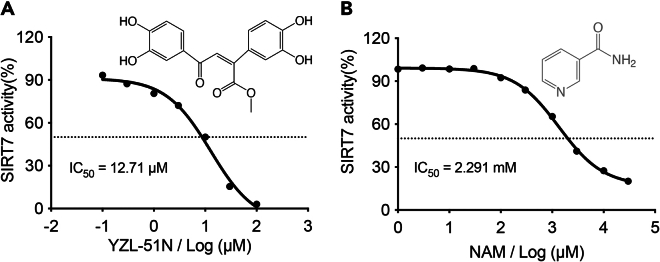
Determination of the IC50 of test compounds (A) Plot shows dose-dependent inhibition of SIRT7 activity by compound **YZL-51N** with an IC_50_ of 12.71 μM. (B) Plot shows dose-dependent inhibition of SIRT7 activity by compound NAM with an IC_50_ of 2.291 mM.

## Expected outcomes

In this protocol, we first outline the large-scale purification of SIRT7 protein, which is suitable for an over 2 L of LB liquid medium cultivation system. Utilizing FPLC system, the yield of the active SIRT7 protein is around 17 mg in 2 L expression culture. The purity of SIRT7 should be more than 95% analyzed by CBB staining after SDS-PAGE (Figure 2). Subsequently, we developed SIRT7 enzyme assay by employing MCA-modified fluorescent peptides. In response to SIRT7 activities, the luminescence responses of the substrate peptides were detected with a linear relationship (y = 31.88x + 2396, R^2^ = 0.9742) (Figure 4). In the first application, we screened the preserved compound library to identify potential SIRT7 inhibitors. As shown in Figure 5, the screening results revealed that compound **YZL-51N** exhibited the most significant inhibitory effect among the tested compounds. Furthermore, we confirmed a dose-dependent inhibition of SIRT7 by compound **YZL-51N**, with an IC_50_ value of 12.71 μM. In contrast, the inhibition caused by NAM was much weaker, with an IC_50_ of 2.291 mM (Figure 6). Therefore, the fluorescent peptide screening technology effectively identified inhibitors of SIRT7.

## Limitations

Fluorescent peptide-based assays have certain advantages in measuring enzyme activities. However, compound autofluorescence is a major issue that can lead to false positive results, severely limiting the drug-screening applications. To address this issue, we first designed a dose-dependent assay to evaluate potential inhibitors and calculate their IC_50_ values. Second, we employed biotin-labeled, non-fluorescent peptide-based assay to confirm the efficacy of the potential compound.^1^ By using these two complementary peptide-based assays, we can effectively address the problem of compound autofluorescence. Additionally, we recognize the potential of advanced alternative technologies, such as time-resolved emission spectroscopy (TRES). This technique can record the luminescence decay profile with high temporal resolution after a pulsed excitation, providing a potential solution to overcome short-lived fluorescent signals from compounds.^5^

## Troubleshooting

### Problem 1

The insufficient purity and low yield of SIRT7 recombinant protein from *E.coli* expression system (related to step 4q in the step-by-step method details).

### Potential solution

The insufficient purity and low yield of SIRT7 protein can be attributed to several factors. To address this issue, we recommend optimizing the following experimental conditions: IPTG concentration, induction temperature, and induction time. Additionally, we have optimized the conditions like buffer composition and column selection (see materials and equipment). If the expected results are still not achieved, consider using alternative fusion tags to enhance protein solubility. These tags, such as Glutathione-S-Transferase (GST) or Maltose-binding protein (MBP), can better to correct protein folding and increase solubility in the *E.coli* cytoplasm.

### Problem 2

The exported fluorescent data show “overflow” from BioTek instrument (related to step 7f in the step-by-step method details).

### Potential solution

An overflow may occur when the fluorescent signal values exceed the maximum limit that can be processed by the BioTek microplate reader. Here are two solutions to this problem: first, reduce the concentration of fluorescent substrate; second, reevaluate the concentrations of other reagents, including SIRT7, NAD^+^ and compounds. Additionally, consider replacing the 96-well white microplate with a black microplate (see key resources table). The white plate enhances the luminescence signal by reflecting light, while black plate reduces signal interference by absorbing fluorescence.

### Problem 3

The absence or inadequacy of fluorescent signals in SIRT7 enzyme assay (related to step 7f in the step-by-step method details).

### Potential solution

The absence or inadequacy of fluorescent signals may be attributed to the poor activity of SIRT7 recombinant protein. To solve this problem, first ensure the quality of SIRT7 protein (refer to problem 1). Additionally, commercially available active SIRT7 protein (BioVision, Cat#7876-50) can serve as a positive control. For peptide handing, aliquot and store peptides at low temperatures: at −20°C for up to one month or at −80°C for up to three months. Furthermore, conduct the reaction in a dark container to prevent any potential photodegradation or interference that may affect the fluorescent signal.

### Problem 4

The fluorescent signal of drug-treated group exceeds than 100% enzymatic activity (related to step 8k in the step-by-step method details).

### Potential solution

There are two possible explanations for this observation: first, the autofluorescence of the compounds may be stronger than the fluorescence generated by the assay system. For potential solutions, refer to the limitations section; second, the compounds may act as activators of the enzyme. To clarify this, additional experiments should be conducted to confirm the activation effect.

### Problem 5

Determining the working concentration of a potential compound in the system (related to step 9f in the step-by-step method details).

### Potential solution

Dose-response relationships are crucial for evaluating drug efficacy. For a drug to elicit a noticeable response, its working concentration in the primary screen should be high enough, depending on the specific circumstances. Additionally, reference the drug concentrations reported in the literature. For dose-dependent verification, design a serial dilution based on the initial dose, typically using 2-fold, 3-fold, or 10-fold serial dilutions. Generally, at least five concentrations should be tested to calculate the IC_50_. Once an IC_50_ value is obtained from the initial test, narrow the concentration range to achieve a more precise IC_50_ value.

## Resource availability

### Lead contact

Further information and requests for resources and reagents should be directed to and will be fulfilled by the lead contact, Wei-Guo Zhu (zhuweiguo@szu.edu.cn).

### Technical contact

Technical questions on executing this protocol should be directed to and will be answered by the technical contact, Wei-Guo Zhu (zhuweiguo@szu.edu.cn).

### Materials availability

This study did not generate any new materials or reagents.

### Data and code availability

This study did not generate any new data or code.

## Acknowledgments

The work was supported by grants from the 10.13039/501100012166National Key Research and Development Program of China (2023YFE0205200), the 10.13039/100014717National Natural Science Foundation of China (82102812, 32090030, and 82273147), the Shenzhen Municipal Commission of Science and Technology Innovation (JCYJ20220818100015032 and RCYX2021070692040047), the Shenzhen Medical Research Fund (B2302010), and the Shenzhen University 2035 Program for Excellent Research.

## Author contributions

W.-G.Z. and T.-S.K. conceived the project and designed the experiments. T.-S.K. and X.L. carried out most of the experiments. J.G. and Y.T. assisted in the protein expression and purification. W.-G.Z. and T.-S.K. analyzed the data and wrote the protocol.

## Declaration of interests

W.-G.Z. and T.-S.K. have a patent registration related to part of this work (patent no. CN113880922B, 2023.06.13).
